# Validation of mDurance, A Wearable Surface Electromyography System for Muscle Activity Assessment

**DOI:** 10.3389/fphys.2020.606287

**Published:** 2020-11-27

**Authors:** Alejandro Molina-Molina, Emilio J. Ruiz-Malagón, Francisco Carrillo-Pérez, Luis E. Roche-Seruendo, Miguel Damas, Oresti Banos, Felipe García-Pinillos

**Affiliations:** ^1^Department of Physical Education and Sports, Faculty of Sport Sciences, Sport and Health University Research Institute (iMUDS), University of Granada, Granada, Spain; ^2^Department of Computer Architecture and Technology, E.T.S.I.I.T.- C.I.T.I.C. University of Granada, Granada, Spain; ^3^Universidad San Jorge, Campus Universitario, Zaragoza, Spain; ^4^Department of Physical Education, Sports and Recreation, Universidad de La Frontera, Temuco, Chile

**Keywords:** wearable, electromyography, knee extension, EMG, validity, muscle contraction, mHealth

## Abstract

The mDurance® system is an innovative digital tool that combines wearable surface electromyography (sEMG), mobile computing and cloud analysis to streamline and automatize the assessment of muscle activity. The tool is particularly devised to support clinicians and sport professionals in their daily routines, as an assessment tool in the prevention, monitoring rehabilitation and training field. This study aimed at determining the validity of the mDurance system for measuring muscle activity by comparing sEMG output with a reference sEMG system, the Delsys® system. Fifteen participants were tested during isokinetic knee extensions at three different speeds (60, 180, and 300 deg/s), for two muscles (rectus femoris [RF] and vastus lateralis [VL]) and two different electrodes locations (proximal and distal placement). The maximum voluntary isometric contraction was carried out for the normalization of the signal, followed by dynamic isokinetic knee extensions for each speed. The sEMG output for both systems was obtained from the raw sEMG signal following mDurance's processing and filtering. Mean, median, first quartile, third quartile and 90th percentile was calculated from the sEMG amplitude signals for each system. The results show an almost perfect ICC relationship for the VL (ICC > 0.81) and substantial to almost perfect for the RF (ICC > 0.762) for all variables and speeds. The Bland-Altman plots revealed heteroscedasticity of error for mean, quartile 3 and 90th percentile (60 and 300 deg/s) for RF and at mean and 90th percentile for VL (300 deg/s). In conclusion, the results indicate that the mDurance® sEMG system is a valid tool to measure muscle activity during dynamic contractions over a range of speeds. This innovative system provides more time for clinicians (e.g., interpretation patients' pathologies) and sport trainers (e.g., advising athletes), thanks to automatic processing and filtering of the raw sEMG signal and generation of muscle activity reports in real-time.

## Introduction

The application of surface electromyography (sEMG) has been widely used to measure muscle activity signals, facilitating access to electrophysiological processes that cause the muscle to generate force and produce movement (De Luca, 1997). In medical and physiotherapy research, sEMG has been used for diagnosis of chronic low back pain patients (Randy Neblett et al., 2014), or the assessment of isokinetic strength testing in patients with posterior cruciate-retaining and substituting knee arthroplasties (Bolanos et al., 1998). In addition, to monitor a physiotherapy exercise programme used as biofeedback for patellofemoral pain syndrome rehabilitation, through isokinetic knee extension strength (Yip and Ng, 2006). Additionally, the muscle activity has contributed to evaluate risk factors, performance and training intervention in sports sciences. For example, data from sEMG have been used to investigate changes in muscle activation patterns of lower limbs in runners wearing different types of footwear (Wakeling et al., 2002), or as a risk factor of injury in football players to prevent the anterior cruciate ligament tear, through isokinetic knee extension strength (De Ste Croix et al., 2015).

The electromyographs present valued benefits providing depth knowledge as an assessment tool in the prevention, monitoring rehabilitation, and training field. The most advanced and complex electromyographs in the field of research have incorporated numerous sEMG channels and synchronization with other technologies such as accelerometry, force platforms and motion capture systems (Bioengineering, 2020; Delsys Inc., 2020). Nevertheless, very few clinicians, physiotherapists and sport professional, working in private rehabilitation or sport centers, are familiar with the sEMG techniques. Traditionally, the prices of electromyographs used in research have been beyond the reach of most clinics, being one of the drawbacks of sEMG techniques. Another drawback, in the private field, is that the muscle activity signal usually needs to be processed and filtered to perform interpretations (De Luca, 1997; De Luca et al., 2010), and some commercial sEMG systems only record the sEMG raw signal without providing software for processing and filtering to the client. This would require preliminary knowledge on programming languages such as Matlab or Python, where programming skills are a must. In addition, it takes time to learn sEMG techniques, to learn these programming skills and to apply the code to process and filter the raw sEMG signal, as well as to create a user-friendly and individualized report for the patient or athlete. Time that private clinics and sports centers cannot afford. Due to the complexity and cost of the equipment, the non-intuitive software and the extensive time to learn and apply signal processing, the assessment of muscle activity becomes a difficult task for non-technical people, thus limiting the ability to make decisions instantly and efficiently.

In recent decades, the technological advances have propelled novel sEMG solutions expanding the range of possibilities in the market. Both traditional and new electromyograph companies are focusing their efforts on the design of portable and lightweight devices. Thus, for example, novel sEMG solutions have been embedded into regular sportswear by integrating electrodes in the textile, although these systems are mostly limited to measuring thigh muscles via sensorized sports tights (Athos, 2020; Myontec, 2020). In addition, some novel sEMG systems have automated their signal processing, generating reports for the patient immediately, hence lowering the complexity that private clinicians, physiotherapists and sport professionals would encounter in traditional neuromuscular analyses (Athos, 2020; BioZen, 2020; Myo, 2020; Myontec, 2020). The mDurance® system (mDurance) is one of such examples, a tool that combines a portable two-channel sEMG device with a mobile application and a cloud service (sEMG signal store, processing and analysis), guiding physiotherapists or sport professionals from placing electrodes by following the European recommendations, until provide a customized report immediately for the patient or athlete. The European recommendations followed by mDurance are part of the SENIAM project (Surface Electromyography for the Non-Invasive Assessment of Muscles) (Hermens et al., 2000). Besides, mDurance allows the possibility to evaluate several muscles along the body given the portable nature of the sensors (Banos et al., 2015; mDurance, 2020). The sEMG device used by mDurance is the Shimmer3 EMG unit (Shimmer), a hardware designed and manufactured by Realtime Technologies Ltd, Dublin, Ireland (Shimmer, 2020). Shimmer is known and used during last decade in engineering research, for being a portable, low-cost and open access device for users with programming skills (Burns et al., 2010a,b; Ahamed et al., 2012; Ibrahim et al., 2016). However, practitioners or coaches could hardly use Shimmer as programming skills are not part of these professional groups. Therefore, the mDurance system is a set made up of Shimmer and its proprietary mDurance software (android app and cloud service), trying to facilitate the sEMG techniques to private rehabilitation and sport centers, guiding its users throughout the neuromuscular assessment process, processing the signal and providing automated reports in real time.

However, all this new technological support must be validated; if not, interpretations after rehabilitation or a medical diagnosis could lead to misinterpretations, what would cause a risk to the patient's health or sports performance. To date, mDurance system, which is hardware (Shimmer) and software (proprietary android app and cloud service), has not been validated yet, so a joint validation (software and hardware) is found fundamental. Hence, the aim of the current study is to evaluate the concurrent validity of the mDurance system, both hardware and software, for measuring muscle activity by comparing sEMG output with a reference sEMG system (i.e., Delsys).

## Materials and Methods

### Participants

Fifteen participants (mean ± SD; age: 27 ± 6 years old; height: 1.76 ± 0.07 m; body mass: 76 ± 13 kg) were recruited for this study. All participants met the inclusion criteria: (1) older than 18 years old; (2) be able to extend and flex the knee with a range of motion >90° (between 90 and 180°); and (3) free of any neurological disorders or musculoskeletal injuries within the last 6 months. After receiving detailed information on the objectives and procedures of the study, each participant signed an informed consent form in order to participate, which complied with the ethical standards of the World Medical Association's Declaration of Helsinki (2013). It was made clear that the participants were free to leave the study at their convenience. The study was approved by the Universidad de La Frontera (Chile).

### Procedures

**Figure 1:**
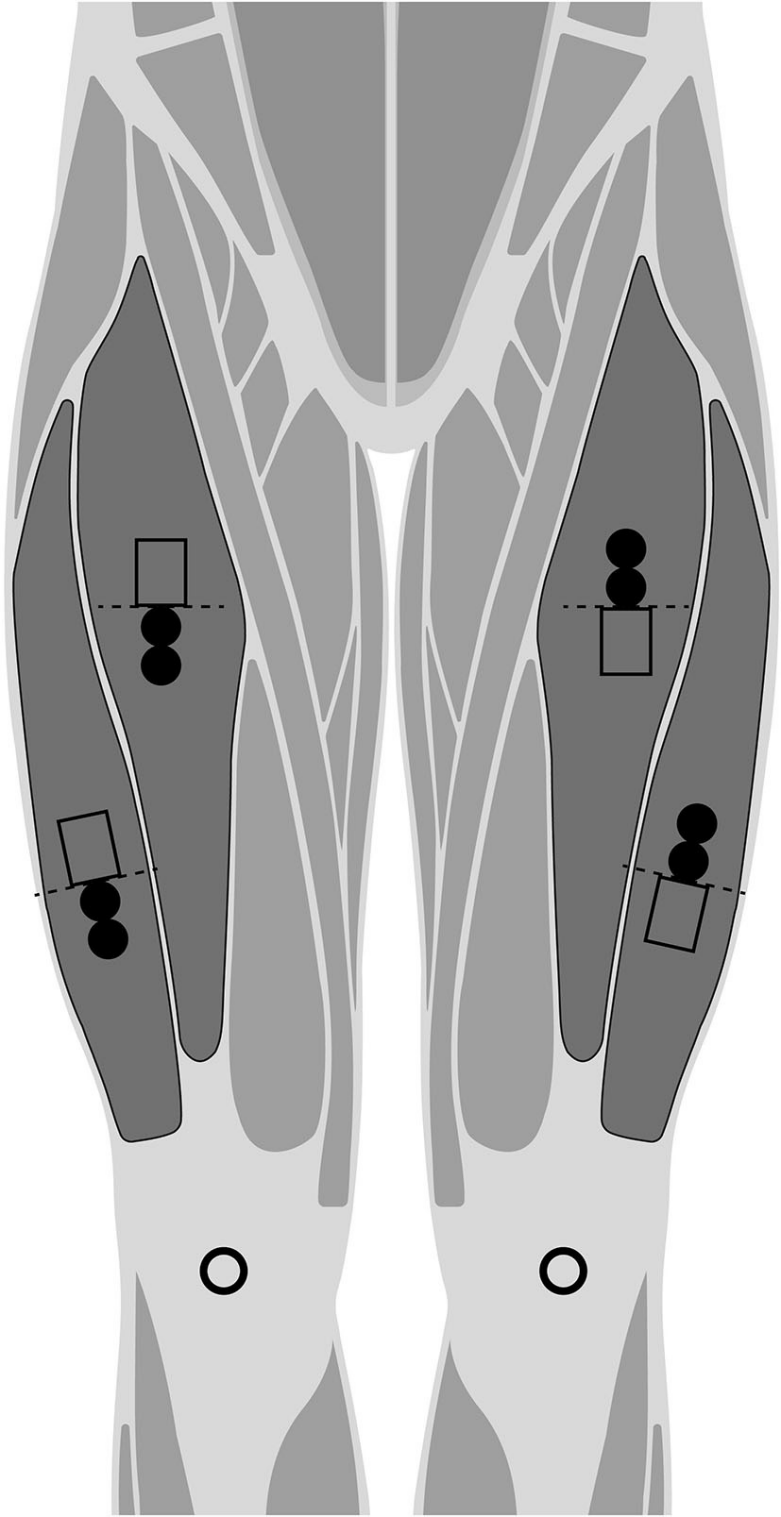
Representation for the placement of the mDurance muscle electrodes (filled circles), and Delsys sensors (bottomless rectangles) to either sides of the SENIAM recommendation (dashed line). Randomized proximal and distal position for mDurance and Delsys in each leg. The mDurance reference electrodes are represented by bottomless circles.

Participants were refrained from severe physical activity for, at least, 48 h before the tests. All tests were conducted, at least, 3 h after eating. Prior to all testing, height and body mass were measured using a precision stadiometer and balance (SECA 222 and 634, respectively, SECA, Corp., Hamburg, Germany) for descriptive purposes. For the measurements, the vastus lateralis (VL) and rectus femoris (RF) were collected from both legs and with both sEMG systems simultaneously (Lynn et al., 2018). The SENIAM project recommendations for skin preparation and electrode location on VL and RF were followed (Hermens et al., 2000). Hence, (1) the skin was shaved if the skin surface was covered with hair (at which the electrodes had to be placed), and (2) was cleaned with alcohol and allowed to vaporize before the electrode was placed. Since this validation is intra-session and during the same muscle contraction, an impedance recording is not required (Elsais et al., 2020). Once the electrode area was found, we marked the place and positioned both mDurance and Delsys electrodes as close as possible to either sides of the mark. Delsys was placed proximally with respect to the mark for the right leg and distally for the left leg; otherwise, mDurance was placed opposite Delsys, distally on the right leg and proximally on the left leg (Figure 1). A reference electrode was placed on the knee at the patella for mDurance, by following its recommendations. Delsys does not require a reference electrode (Figure 1).

### Material and Testing

For the validation, two-different sEMG systems were used: mDurance, as a novel sEMG system, and Delsys TrignoTM Wireless, as a reference system:

- The mDurance® system (mDurance Solutions SL, Granada, Spain) is a portable and low-cost sEMG system that consists of three parts. First, a Shimmer3 EMG unit (Realtime Technologies Ltd, Dublin, Ireland), that is a bipolar sEMG sensor for the acquisition of superficial muscle activity. Each Shimmer sensor is composed of two sEMG channels, with a sampling rate at 1,024 Hz. Shimmer applies a bandwidth 8.4 kHz, the EMG signal resolution is 24 bits and overall amplification of 100–10,000 V/V. The electrodes used were pre-gelled Ag/AgCl with a diameter of 10 mm and an interelectrode distance of 20 mm. Second, the mDurance (Android) mobile application is responsible for receiving data from the Shimmer unit and sending it to a cloud service. And, third, the mDurance cloud service where the sEMG signals are stored, filtered, and analyzed, generating the reports.
- TrignoTM Wireless System (Delsys, Inc., Natick, MA, USA), henceforth Delsys, was the reference system used to compare with the novel electromyograph. It is a widespread commercial sEMG system with 16 channels and a sampling rate at 2,000 Hz. Besides, Delsys applies a built-in filter of 20–450 Hz bandwidth, the EMG signal resolution is 16 bits, and the inter-sensor latency <500 us. The acquisition was carried out via the EMGworks® Software (Delsys, Inc., in Natick, MA., USA).

Both sEMG systems were compared through their natural hardware configurations despite their differences (i.e., sampling rate, bandwidth filter), and it was taken into account for the processing and filtering of the signal.

A HUMAC Norm (CSMi, Inc., Stoughton, MA, USA) isokinetic dynamometer was used to control and measure angular displacement during knee extension sets. The dynamometer was used to reduce variability in the performance of the movement by controlling motion speed and position.

**Figure 2:**
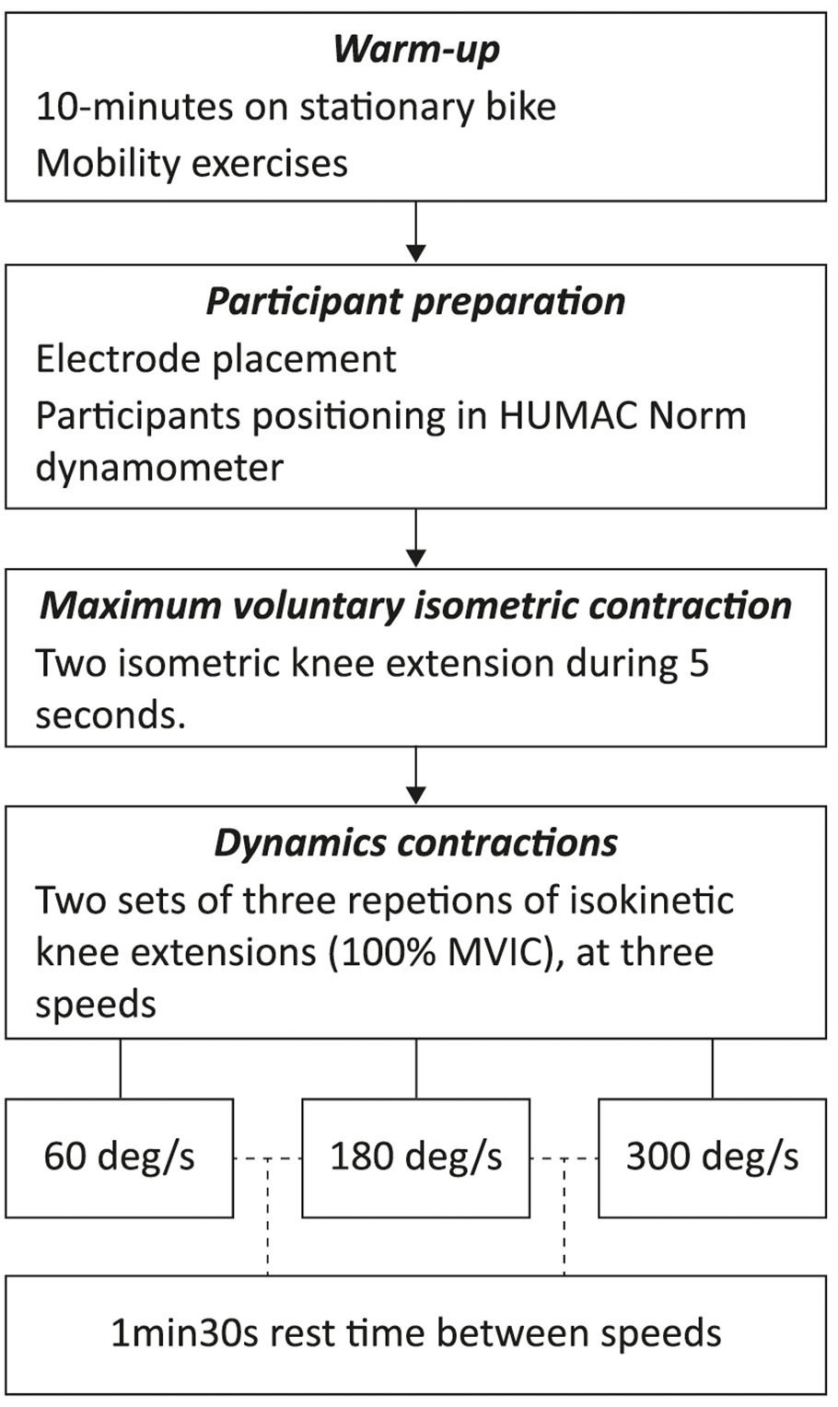
Overview diagram of the validation testing.

For the validation testing, participants were instructed to cycle for 10-min on a stationary bike at a self-selected pace followed by mobility exercises (e.g., dynamics drills) for the warm up (Figure 2). Then, we carried out the placement of the electrodes and participants were then seated on the HUMAC Norm dynamometer (Figure 2). Then, they were positioned according to the HUMAC testing and rehabilitation user's manual (CSMi Solutions, 2013): hip angle at 85°, knee flexed at 90°, and the ankle in the anatomical position (sole of the foot perpendicular to the shank). The padded arm of the dynamometer was positioned 3 cm proximally to the lateral malleolus and the axis of rotation of the knee aligned with the axis of rotation of the dynamometer (Kay and Blazevich, 2009). This position was used to carry out the maximum voluntary isometric contraction (MVIC) for two repetitions of the knee extension isometric test during 5 s, separated by a 2 min rest period (Roberts et al., 2012; Luc et al., 2016) (Figure 2). In addition, this position was maintained during all subsequent dynamic tests and familiarization repetitions were included in all tests with each speed and movement (Figure 2). Following MVIC testing, participants performed two sets with three repetitions of concentric knee extension at 100% of intensity for each testing speed: 60, 180, and 300 deg/s (Lynn et al., 2018). No repetitions at sub-maximal intensity were performed to avoid failed attempts, thus reducing the risk of adding potential variables that influence the SEMG signal (such as accumulated fatigue or increased sweating). Three repetitions is a reliable amount to maintain the intensity between contractions and sets (Sole et al., 2007). Between sets, a 1 min and 30 s rest period was defined. Besides, during the concentric knee flexion, the isokinetic dynamometer did not offer any resistance to be useful as a rest period between repetitions (Lynn et al., 2018). The dynamic tests were carried out unilaterally, so all the sets of one leg were performed and followed by the other leg separately, with randomized order.

### Signal Processing and Filtering

For the processing and filtering of the raw signals of the two independent systems into the sEMG output signal, we implemented in Python the mDurance methodology used in its cloud service. (1) First, both the isometric and dynamic tests were filtered using a fourth order Butterworth bandpass filter with a cut-off frequency at 20–450 Hz, only for mDurance raw signal since Delsys already includes this filter by the hardware. (2) Second, the signal was smoothed using a window size of 0.025 s root mean square (RMS) and a overlapping of 0.0125 s between windows for both systems separately. (3) And third, the MVIC value was calculated using the average of the three maximum peaks of the RMS signal during the 5 s isometric test, and being the MVIC used for normalized the RMS signal of each system. The use of the average (following mDurance mythology) instead of the peak for the MVIC calculation will not affect the magnitude of the sEMG outputs inter-systems (Burden, 2010). Python libraries used for the analysis were Numpy (Van Der Walt et al., 2011) for numerical computations, Scipy (Virtanen et al., 2020) for signal analysis and Pandas (McKinney, 2010) for processing the CSV files that contained the raw sEMG.

**Figure 3:**
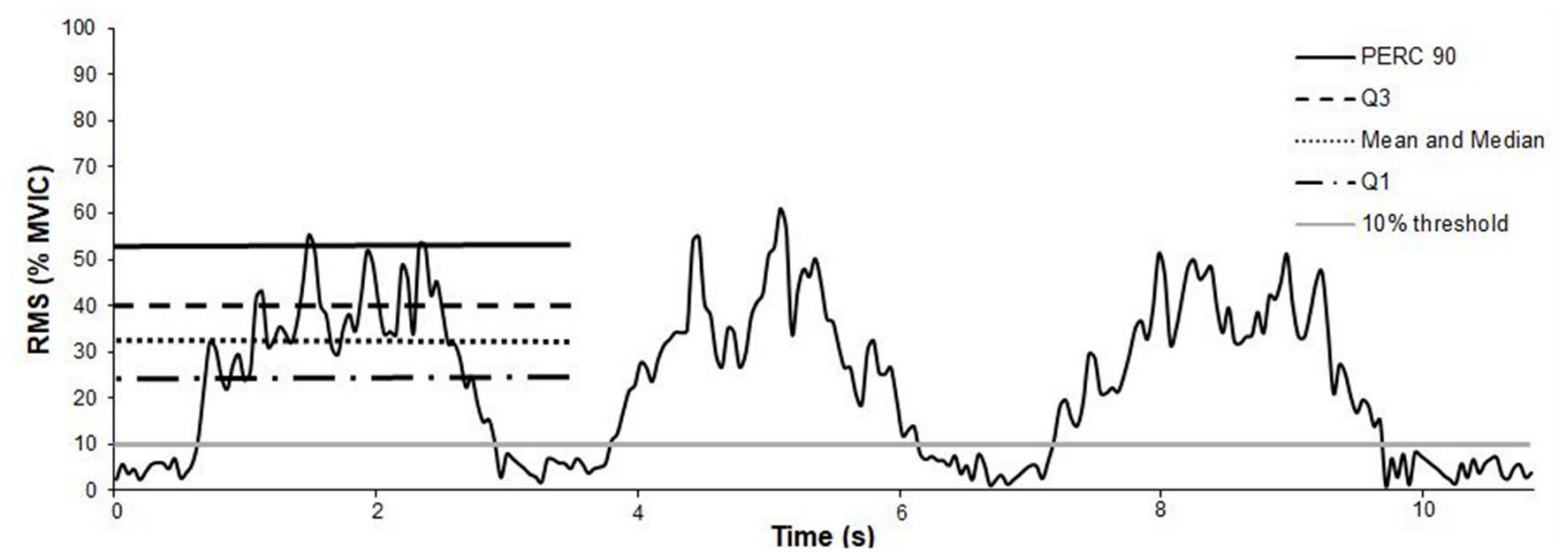
Representation of the 10% threshold (gray horizontal solid bar) applied to an exemplary set, with a root mean square (RMS) signal normalized by the maximum voluntary isometric contraction (MVIC). Besides, representation of the muscle activity parameters for an exemplary muscle contraction. sEMG amplitude parameters: 90th percentile (PERC 90), third quartile (Q3), mean and median, and first quartile (Q1).

To determine the onset and offset of each muscle contraction, a threshold of 10% was applied to the normalized RMS signal from the MVIC (Figure 3) (De Luca, 1997). Subsequently and for the analysis of sEMG amplitude signals, we calculated the following variables of each contraction for each sEMG system separately: mean, median, first quartile (Q1), third quartile (Q3) and 90th percentile (PERC90). These metrics represent the different levels of the sEMG amplitude signal for each muscle contraction: (i) PERC90 represents the highest level of the contraction, (ii) Q3 is the next level, (iii) median and mean that represents the middle level of the muscle contraction and, (iv) Q1 represents the lowest level of the contraction (Figure 3). Finally, the average of each set executed with three useful contractions in all variables was calculated (i.e., mean, Q1, Q3, or PERC90), as sample for statistical analysis in this concurrent validation. The inclusion criteria for a useful contraction was established at a baseline noise below 15 μVrms and a correct execution of the participant respecting the contraction-rest times. These inclusion criteria were used to decide the useful sets for the statistical analysis. All sEMG outputs were based on the RMS amplitude since mDurance have no integrated a sEMG frequency analysis (i.e., Fourier transform).

### Statistical Analysis

A total of thirty-eight sets were used, which correspond to the number of useful series that met the inclusion criteria. Data analysis was conducted using SPSS (vs. 25, SPSS Inc., Chicago, IL). Descriptive statistics are represented as mean and standard deviation (SD). In order to examine the concurrent validity between systems (i.e., mDurance vs. Delsys), the level of agreement between sEMG data from both devices was examined. To that end, a comparison of means (*t*-test) was conducted between systems, and the Cohen's *d* effect size (ES) was used to interpret the magnitude of the differences (Cohen, 1988) (i.e., trivial ES < 0.19, small 0.2–0.49, medium 0.5–0.79, and large ES ≥ 0.8). A Pearson correlation analysis was also conducted. To properly interpret the magnitude of the correlations, the following criteria were adopted: <0.1 (trivial), 0.1–0.3 (small), 0.3–0.5 (moderate), 0.5–0.7 (large), 0.7–0.9 (very large), and 0.9–1.0 (almost perfect) (Hopkins et al., 2009). The validity analysis was reinforced by calculating the intra class correlation coefficients (ICCs) between mDurance and the reference system. Based on a previous study (Koo and Li, 2016), the authors conducted a two-way random-effects model (ICC [2,k]), mean of measurements and absolute definition for the ICCs. The magnitude of the ICC was interpreted according to the following benchmarks (Landis et al., 1977): ICC <0 (poor), 0–0.20 (slight), 0.21–0.40 (fair), 0.4–0.60 (moderate), 0.61–0.80 (substantial), and >0.80 (almost perfect). Finally, Bland-Altman plots, based on the limits of agreement method (Bland and Altman, 1995), let us examine the presence of systematic and proportional bias between systems. Heteroscedasticity of error was defined as an *r*^2^ > 0.1 (Atkinson and Nevill, 1998). The level of significance used was *p* < 0.05.

## Results

Despite the pairwise comparisons showed some significant differences (*p* < 0.05) between systems for measuring the sEMG activity of the RF at different speeds (Table 1), the ES resulted trivial and small differences in all cases. The Pearson correlation analysis reported very large relationships (*r* > 0.7) between data from both systems for all parameters at 60, 180, and 300 deg/s. The ICCs reported almost perfect relationships (ICC > 0.81) for every variable at any speed condition (60, 180, and 300 deg/s).

**Table 1:** Concurrent validity (i.e., Delsys vs. mDurance systems) of RMS normalized for the RF muscle at different speeds of movement (i.e., 60–180–300 deg/s).

**Speed**	**Variable**	**Delsys**	**MDurance**	***P*-value (Cohen's *d*)**	**Pearson coefficient (*r*)**	**ICC (95% CI)**
60 deg/s	Mean	47.36 (12.47)	49.30 (10.28)	0.045 (0.17)^*^	0.890^***^	0.927 (0.855–0.963)
	Median	47.08 (13.38)	49.24 (11.54)	0.057 (0.17)	0.862^***^	0.915 (0.833–0.956)
	Q1	33.53 (10.59)	35.17 (8.97)	0.116 (0.17)	0.806^***^	0.882 (0.773–0.939)
	Q3	60.04 (16.53)	63.11 (13.80)	0.039 (0.20)^*^	0.844^***^	0.900 (0.801–0.949)
	PERC90	72.05 (19.87)	75.64 (16.16)	0.058 (0.19)	0.823^***^	0.885 (0.776–0.941)
180 deg/s	Mean	44.91 (13.90)	46.73 (12.01)	0.149 (0.14)	0.819^***^	0.893 (0.801–0.942)
	Median	46.73 (12.01)	44.93 (14.91)	0.286 (0.13)	0.807^***^	0.890 (0.796–0.941)
	Q1	27.15 (9.11)	28.85 (10.63)	0.112 (0.16)	0.774^***^	0.862 (0.744–0.926)
	Q3	60.30 (20.64)	62.80 (17.60)	0.227 (0.13)	0.771^***^	0.863 (0.747–0.926)
	PERC90	73.72 (25.15)	76.60 (19.52)	0.295 (0.13)	0.717^***^	0.819 (0.666–0.903)
300 deg/s	Mean	42.23 (14.17)	44.08 (11.30)	0.131 (0.15)	0.835^***^	0.895 (0.805–0.943)
	Median	40.53 (14.44)	43.22 (12.83)	0.024 (0.19)^*^	0.858^***^	0.912 (0.829–0.954)
	Q1	24.60 (8.90)	26.54 (9.15)	0.049^*^ (0.21)^*^	0.763^***^	0.857 (0.731–0.924)
	Q3	57.91 (20.79)	60.21 (15.36)	0.236 (0.13)	0.807^***^	0.870 (0.759–0.930)
	PERC90	71.79 (26.49)	72.67 (18.23)	0.747 (0.22)	0.749^***^	0.827 (0.677–0.907)

*RMS, root mean square; RF, rectus femoris; Q1, quartile 1; Q3, quartile 3; PERC90, 90th percentile*.

* denotes p < 0.05;

*** *denotes p < 0.001*.

Regarding the other muscle (i.e., VL), the means comparison revealed the lack of differences at any speed (Table 2). The correlation analysis reported significant correlation with large to very large Pearson coefficients (*r* > 0.609, *p* < 0.001), and substantial to almost perfect ICCs (ICC > 0.762) for all parameters at every speed.

**Table 2:** Concurrent validity (i.e., Delsys vs. MDurance systems) of RMS normalized for the VL muscle at different speeds of movement (i.e., 60–180–300 deg/s).

**Speed**	**Variable**	**Delsys**	**MDurance**	***P*-value (Cohen's *d*)**	**Pearson coefficient (*r*)**	**ICC (95% CI)**
60 deg/s	Mean	46.15 (11.76)	45.51 (10.80)	0.666 (0.06)	0.721^***^	0.840 (0.671–0.922)
	Median	45.88 (12.74)	44.85 (11.36)	0.524 (0.09)	0.729^***^	0.842 (0.677–0.923)
	Q1	31.98 (9.75)	31.83 (9.52)	0.899 (0.01)	0.768^***^	0.872 (0.737–0.938)
	Q3	59.32 (15.73)	58.55 (14.08)	0.715 (0.05)	0.691^***^	0.818 (0.626–0.911)
	PERC90	71.91 (19.38)	70.30 (16.85)	0.561 (0.09)	0.642^***^	0.781 (0.550–0.892)
180 deg/s	Mean	46.77 (14.57)	45.23 (12.91)	0.310 (0.12)	0.779^***^	0.872 (0.756–0.933)
	Median	44.04 (14.97)	43.47 (13.58)	0.682 (0.04)	0.824^***^	0.903 (0.813–0.950)
	Q1	27.10 (10.06)	27.71 (9.93)	0.526 (0.06)	0.832^***^	0.910 (0.827–0.953)
	Q3	63.26 (20.58)	60.39 (19.18)	0.250 (0.14)	0.715^***^	0.831 (0.677–0.912)
	PERC90	79.26 (26.67)	75.68 (22.66)	0.196 (0.15)	0.681^***^	0.801 (0.619–0.896)
300 deg/s	Mean	48.40 (15.62)	47.17 (13.17)	0.387 (0.15)	0.856^***^	0.916 (0.831–0.958)
	Median	46.86 (16.72)	46.28 (14.25)	0.711 (0.17)	0.847^***^	0.913 (0.824–0.957)
	Q1	26.45 (7.87)	26.85 (7.72)	0.741 (0.05)	0.609^***^	0.762 (0.516–0.883)
	Q3	67.04 (24.26)	64.03 (20.05)	0.214 (0.13)	0.827^***^	0.895 (0.789–0.948)
	PERC90	83.40 (30.51)	80.51 (24.00)	0.351 (0.11)	0.818^***^	0.886 (0.771–0.944)

*RMS, root mean square; VL, vastus lateralis; Q1, quartile 1; Q3, quartile 3; PERC90, 90th percentile*.

*** *denotes p < 0.001*.

In order to ensure the lack of effect of electrode position (i.e., proximal vs. distal), a between systems comparison (i.e., Delsys vs. MDurance) was performed for every parameter at every speed for both right and left leg. Almost perfect ICCs were obtained, with ICCs > 0.88, where the confidence interval spans between moderate and almost perfect (0.411–0.980) in all cases.

**Figure 4:**
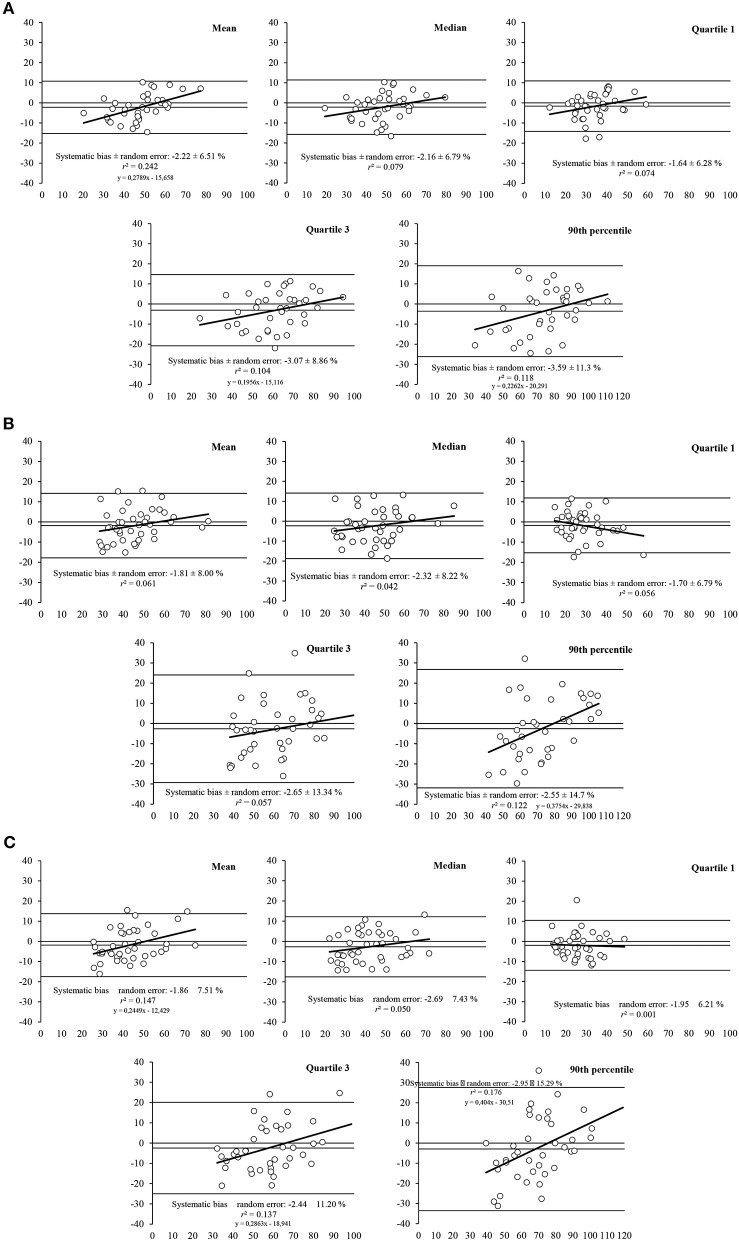
Bland-Altman plots for the measurement of muscle activity parameters (i.e., mean, median, first quartile, third quartile, and 90th percentile) for the RF muscle at different speeds of movement: **(A)** 60 deg/s, **(B)** 180 deg/s, and **(C)** at 300 deg/s. The plots includes the mean difference (dotted line) and 95% limits of agreement (dashed lined), along with the regression line (solid line).

The Bland-Altman plots revealed heteroscedasticity of error for some sEMG-related parameters of the RF at 60 deg/s (i.e., mean, Q3, and PERC90), at 180 deg/s (i.e., PERC90), and 300 deg/s (i.e., mean, Q3, and PERC90) (Figure 4).

**Figure 5:**
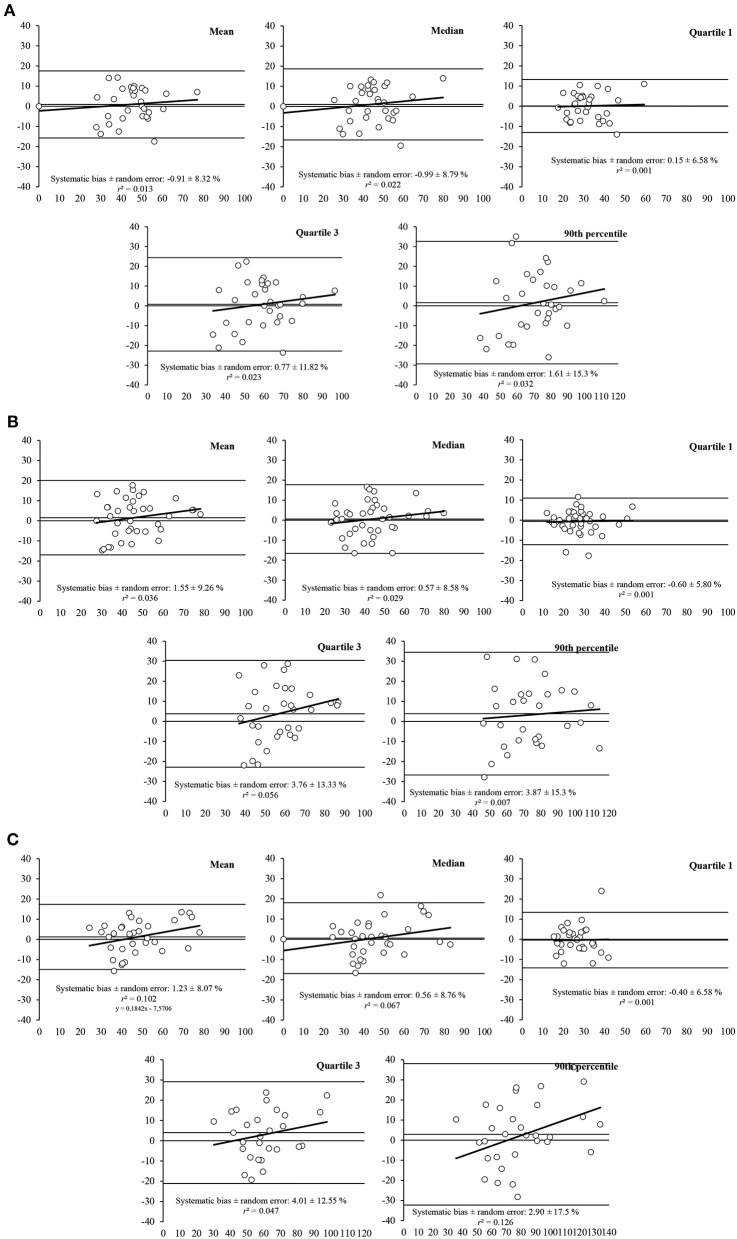
Bland-Altman plots for the measurement of muscle activity parameters (i.e., mean, median, first quartile, third quartile, and 90th percentile) for the VL muscle at different speeds of movement: **(A)** 60 deg/s, **(B)** 180 deg/s, and **(C)** at 300 deg/s. The plots includes the mean difference (dotted line) and 95% limits of agreement (dashed lined), along with the regression line (solid line).

For the VL (Figure 5), the Bland-Altman plots also revealed heteroscedasticity of error for mean and PERC90 at 300 deg/s, whereas no heteroscedasticity was found in any sEMG parameter at 60 or 180 deg/s.

## Discussion

This study aimed at determining the validity of the mDurance system for measuring muscle activity during isokinetic knee extensions at three different speeds (i.e., 60, 180, and 300 deg/s), for two muscles (i.e., RF and VL) and two different electrodes locations (i.e., proximal and distal placement), by comparing sEMG output with a reference sEMG system, the Delsys. The results obtained suggest that the mDurance system provides valid sEMG output data for VL and RF during knee extensions at 60, 180, and 300 deg/s speed, showing a strong concurrent validity as compared to Delsys sEMG system, based on Pearson's coefficients and ICCs referring to a relative validity. However, Bland-Altman plots suggest an acceptable absolute validity, due to the random errors, going from ±8% for the mean to ±15% for the Perc90.

The main use of the mDurance is the muscle assessment for clinics and sports professionals, due to its low price, low weight and automated signal processing. Typical situations to use mDurance could be the clinical evaluation of low back pain through the endurance test (Banos et al., 2015) or the flexion-relaxation phenomenon (Carrillo-Perez et al., 2018), and the neuromuscular assessment of the curve sprint in football at high intensity, in the field of sport biomechanics (Filter et al., 2020). Nevertheless, the use of a non-validated novel sEMG system could be harmful to the health of patients or affect the performance of athletes, due to potential misinterpretations. Regarding relative validity, the ICCs where reported an almost perfect relationship for the VL (ICC > 0.81) and substantial or almost perfect relationship for the RF (ICC > 0.762), for all analyzed variables. Another interesting result has been a very large relationship (*r* > 0.7, *p* < 0.001) reported by the Pearson correlation analysis for the RF, and significant for the VL (*r* > 0.609, *p* < 0.001), for all speeds and all variables. In comparison with our results, Burns et al. (2010a) compared Shimmer with a commercial EMG system (Grass P511AC Amplifier system) in biceps brachii muscle during isometric contractions. They found a variation for the mean of approximately 8%, between systems. On the other hand, our results revealed a lack of differences at any speed for VL with 1–2% mean differences approximately and some differences for RF, being the effect size trivial in all cases, with 2–3% mean differences approximately.

We suggest that although RF and VL are part of the quadriceps and the longitudinal muscles, the slight differences found could be caused by slight anatomical differences between them. The anatomical differences could be related to the location of the muscle in relation to the quadriceps, the proximity of other muscles, tendons or ligaments affecting crosstalk (Blanc and Dimanico, 2014), and the difference in size for the optimal electrode placement site which is greater for RF (Hogrel et al., 1998; Barbero et al., 2012). Besides, a prior work showed that the RF muscle is regionally non-uniformly activated longitudinally (Watanabe et al., 2014).

A similar study compared a textile sEMG system with a traditional electromyograph for the quadriceps musculature in isokinetic knee extensions at 60, 180, and 300 deg/s (Lynn et al., 2018). Similar to our results, they observed that both systems had no significant differences for all of the computed variables. Moreover, their Pearson's coefficients showed a similar magnitude and directionality of correlation for both systems, without a significant inter-system difference. For instance, they found correlations between 0.69 and 0.74 for all their muscles at the speeds of 60 and 300 deg/s, which indicated a very large agreement between both. In the same way, a prior work compared a customized sEMG system also with Delsys, although using dynamic exercises (knee extension, squat, lunge, and jump). Their findings indicated a good to excellent agreement between both systems (Fuentes del Toro et al., 2019), with correlations between 0.51 and 0.96 in their results, with a mean of 0.60. Although, different types of correlation were performed, we recommend caution when establishing comparisons between studies. In comparison with our findings, we observed for the Pearson correlation analysis a result between 0.72 and 0.89 for the RF and between 0.61 and 0.83 for the VL. This represents a very large ratio for each muscle for the concurrent validation between mDurance and Delsys. Hence, we found similar results with significant and very large Pearson's correlation between mDurance and Delsys. Lastly, although the signal processing and the parameters obtained between Fuentes del Toro et al. (2019) and Lynn et al. (2018) are different from ours, they are comparing a reference system with a new one like we do here, and their findings and ours are quite similar, obtaining good and excellent agreements between both systems.

Regarding the absolute validity, the Bland–Altman plots evaluate the agreement between the mDurance and Delsys. Our plots for the RF revealed heteroscedasticity for the mean, Q3, and Per90 at 60 and 300 deg/s and only Per90 at 180 deg/s. Although for VL muscle, we only found heteroscedasticity for mean and Per90 at 300 deg/s. The heteroscedasticity refers to the relationship between the difference in the two methods and the size of the measured variable. In addition, the systematic bias for the mean was −2% with a random error of ±7.34%, and 1.23 ± 8.55% random error, for RF and VL, respectively and for all speeds. Being a good-enough systematic bias and random error for clinic assessments as the flexion-relaxation phenomenon associated with chronic low back pain (Alschuler et al., 2009) and testing the efficacy of sEMG biofeedback in knee osteoarthritis by knee extensions at 60 and 180 deg/s (Yilmaz et al., 2010). However, the Per90, which represents the top of the muscle contraction, showed a systematic bias for all speeds of 3.03% and a random error of ±13.76% for RF, and 2.8 ± 16.03%, being an acceptable systematic bias and random error in this context. This may result because sEMG is more variable as closer to the peak (Hibbs et al., 2011). In this direction, the random error was wider at higher speeds for all variables in both muscles. For example, Perc90 at 60 deg/s showed a random error of ±15.3%, however at 300 deg/s was ±17.5%.

All our findings indicate that there is an excellent agreement between mDurance and Delsys, although it should be noted that there are various limitations to this study. Firstly, all the tests were carried out at 100% of the MVIC, aiming to not make extra attempts, and avoid causing fatigue because of it. Limiting ourselves to not having information about the effect of fatigue on the different systems. Secondly, in order to create a controlled environment, only the quadriceps musculature and isokinetic knee extensions were assessed. Therefore, for future studies it is recommended to check the validity of other musculature in addition to checking more functional tests for clinical (i.e., lumbar flexion for low back pain patients or gait) and sports (i.e., running, squats, or jumps) applications. Thirdly, the lack of standardized methods and analysis for sEMG validations studies may make difficult to compare with similar validations. Finally, an important consideration is that validation data were obtained from an analysis based on within-participant variation (CV) rather than on different days (i.e., test-re-test) so, our current reliability statistics might not generalize to runs performed several days apart.

## Conclusions

The results indicate that the mDurance® sEMG system is a valid tool to measure muscle activity during isokinetic contractions over a range of speeds, tested in laboratory conditions. Our results revealed an excellent relative validity for all variables, and an acceptable to good-enough absolute validity, with a systematic bias of 1.62 ± 8% random error for the mean. From a practical standpoint, mDurance as a system made up of Shimmer sensor and proprietary software, is a valid sEMG system, as compared to Delsys. This novel system includes a user-friendly software, a light hardware and a reasonable price, which make it more affordable and accessible for clinicians and sport trainers. In addition, it provides more time for interpretation and for addressing patients' pathologies or making recommendations to athletes instantly, thanks to the generation of real-time reports. Further research is required to establish reliability (test-re-test) over several days and to check the validity of mDurance measurements in more muscles from other areas and sizes, in more exercises modalities (i.e., walking, running, cycling) and at different submaximal intensities.

## Data Availability Statement

The raw data supporting the conclusions of this article will be made available by the authors, without undue reservation.

## Ethics Statement

The studies involving human participants were reviewed and approved by Universidad de La Frontera, Temuco, Chile. The patients/participants provided their written informed consent to participate in this study.

## Author Contributions

AM-M, FC-P, MD, and OB defined the experimental design. AM-M and ER-M collected the data. FC-P carried out in Python the mDurance's cloud computing simulation for signal processing and filtering. FG-P carried out the statistical analysis. AM-M conceptualized the approach and together with FG-P wrote the paper. LR-S, MD, OB, ER-M and FG-P reviewed the manuscript for scientific content. All authors have read and agreed to the published version of the manuscript.

## Conflict of Interest

MD and OB were affiliated with the company mDurance Solutions S.L. The remaining authors declare that the research was conducted in the absence of any commercial or financial relationships that could be construed as a potential conflict of interest.
